# Design and application of implicit solvent models in biomolecular simulations

**DOI:** 10.1016/j.sbi.2014.04.003

**Published:** 2014-04

**Authors:** Jens Kleinjung, Franca Fraternali

**Affiliations:** 1 Division of Mathematical Biology, MRC National Institute for Medical Research, The Ridgeway, London NW7 1AA, United Kingdom; 2 Randall Division of Cell and Molecular Biophysics, King's College London, New Hunt's House, London SE1 1UL, United Kingdom

## Abstract

We review implicit solvent models and their parametrisation by introducing the concepts and recent devlopments of the most popular models with a focus on parametrisation *via* force matching. An overview of recent applications of the solvation energy term in protein dynamics, modelling, design and prediction is given to illustrate the usability and versatility of implicit solvation in reproducing the physical behaviour of biomolecular systems. Limitations of implicit modes are discussed through the example of more challenging systems like nucleic acids and membranes.

## Highlights

• Implicit solvent replaces explicit water by a potential of mean force.
• Popular models are SASA, VOL and Generalized Born.
• Implicit solvent is used in MD, protein modelling, folding, design, prediction and drug screening.
• Large-scale simulations allow for parametrisation *via* force matching.
• Application to nucleic acids and membranes is challenging.

**Current Opinion in Structural Biology** 2014, **25**:126–134

This review comes from a themed issue on **Theory and simulation**

Edited by **Rommie E Amaro** and **Manju Bansal**

For a complete overview see the Issue and the Editorial

0959-440/© 2014 The Authors. Published by Elsevier Ltd. This is an open access article under the CC BY license (http://creativecommons.org/licenses/by/3.0/)

**http://dx.doi.org/10.1016/j.sbi.2014.04.003**

## Introduction

It has been 37 years since the first molecular dynamics (MD) simulations of the protein BPTI *in vacuo* has been published [1]. In the meantime the technique has progressed appreciably in applicability and efficiency. Three of the founders of the field, namely Martin Karplus, Michael Levitt and Arieh Warshel, were awarded the Nobel Prize in Chemistry 2013 for the “development of multi-scale models of complex chemical systems” [2,3]. Within a few decades MD simulations have become an essential and complementary tool for the structural and conformational study of biomolecules [4]. Nevertheless, efficient and accurate multi-scale modelling of complex biochemical systems remains a challenging task.

Many central aspects of biomolecular simulations have undergone rapid advancements: the reproducibility of a biomolecular system's representation by classical force fields, the combined treatment of reactions and dynamics *via* QM/MM calculations and the use of coarse-grained models for very large systems.

**Figure 1:**
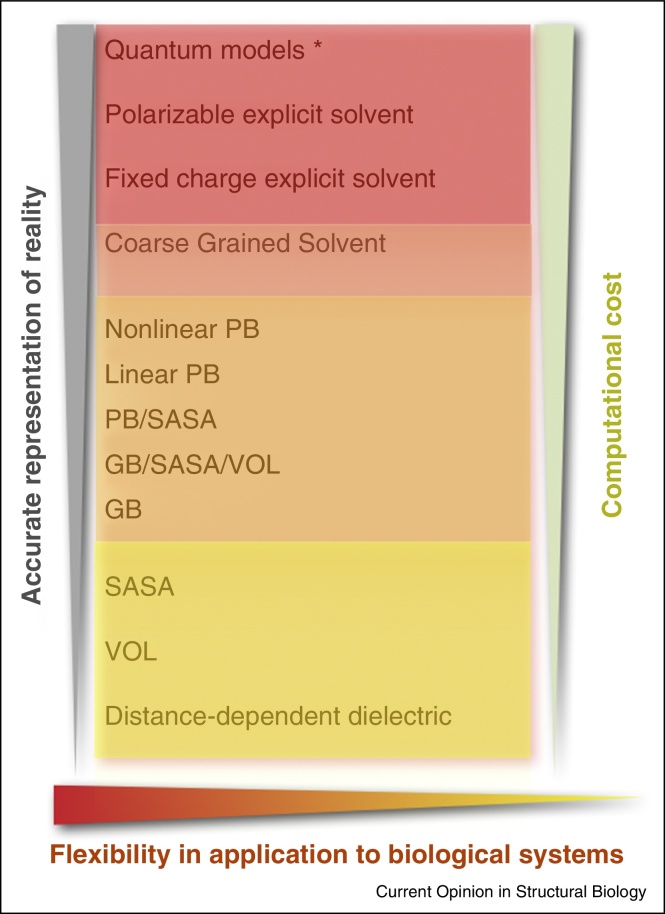
Implicit solvation models.

Multi-scale approaches are designed to bridge spatial and temporal scales by merging models with different resolution. The underlying physical descriptions can also vary with scale: first, the quantum mechanical treatment of degrees of freedom at (sub-)atomic scales, second, the classical representation of discrete atoms, molecules or groups of molecules at intermediate scales, and third, the mesoscopic or continuum treatment of entire molecular systems (Figure 1).

A multi-scale model should be able to balance accuracy and efficiency and integrate techniques with different resolution to provide a realistic description of the system's behaviour. A good molecular model can be directly used for experimental testing and/or validation. Interestingly, one of the first methods in the spirit of multiscaling was Warshel's pioneering approach to the development of solvent models [5].

Modelling water is essential for the description of cellular biopolymers, as they have evolved to function in an environment containing predominantly water, enriched with ions, proteins, lipids and sugars. The description of the polyfunctional nature of each of these components is intrinsically difficult and can only be partially formalised for *in silico* applications. Most biomolecular simulations account for the presence of the native (cellular) environment either explicitly, for example by inclusion of water molecules and ions, or implicitly, by approximating the mean force (Potential of Mean Force, PMF) exerted by the external media on the biomolecule. Implicit water models can be considerably faster to compute, because the implicit solvent contributes no or few degrees of freedom to the simulation. However, they neglect specific important features such as hydrogen bond fluctuations at the solute surface, water dipole reorientation in response to conformational changes and bridging water molecules. Generally implicit solvent tends to be a good approximation where it models isotropic and bulk solvent.

To sample efficiently the dynamics of a system with many degrees of freedom or along large time scales, coarse-graining by bundling degrees of freedom into pseudo-atoms is a commonly used technique. This transformation smoothens the underlying energy hyper-surface and speeds up considerably energy and force calculations [6–9]. Typical strategies for multi-scaling are capturing the physical effects of the presence of solvent *via* an intrinsic term that is directly related to the solvation free energy of transfer of a rigid solute from vacuum to solution or by parametrising the implicit solvent by matching solute properties like explicit water forces or conformational ensembles [11^••^,12^••^]. In recent years, large-scale atomistic simulations in different types of solvent have been performed and the trajectories have been made publicly available [13–15]. These data can be fruitfully used for the parametrisation of coarse-grained models and implicit solvent models.

Some simplified and fast implicit solvation models use a first-shell approximation of the solvent effect, under the assumption that the forces exerted on a solute atom by the solvent are proportional to the solvent-accessible surface area (SASA) of the solute atom. This assumption holds for the nonpolar part of the solute-solvent interactions, which is complemented by a contribution that models electrostatic interactions, on the basis of the approximation that the bulk solvent behaves as a dielectric continuum. The electrostatic term is either integrated into the parametrisation of the SASA model or treated by a separate energy term.

Previous reviews on implicit solvent models have been published in this journal [16–18] and in others [19–23]. We will first briefly introduce the most frequently used models and then focus on parametrisations *via* force matching and applications that require fast estimates of the free solvation energy.

## Models of implicit solvation

### Free energy of solvation

The free energy of solvation is traditionally decomposed into three components that relate to a purely theoretical process: creation of a cavity within the solvent to accommodate the solute molecule (Δ*G*_*cav*_) and embedding of the solute molecule into the cavity leading to van der Waals (Δ*G*_*vdW*_) and electrostatic (Δ*G*_*ele*_) interactions between solute and solvent.

(1) $$\Delta G_{sol}=\Delta G_{cav}+\Delta G_{vdW}+\Delta G_{ele}$$

Solvent-Accessible Surface Area methods model either the nonpolar terms Δ*G*_*cav*_ + Δ*G*_*vdW*_ or the entire Δ*G*_*sol*_ term, depending on their parametrisation. Poisson–Boltzmann and Generalized-Born methods model the Δ*G*_*ele*_ term.

### Solvent-accessible surface area (SASA) models

The first SASA parametrisations were performed by Eisenberg and McLachlan on the basis of the free energy of transfer of amino acids between octanol and water [10^••^] and by Ooi *et al.* on seven chemical groups whose thermodynamic solvation parameters were derived from a benchmark set of small molecules [24^•^].

Implicit solvation models on the basis of SASA assume the interactions between solute and solvent to be proportional to the surface area. Therefore, the free energy of solvation Δ*G*_*sol*_ of a solute molecule is described by a mean solvation potential $V_{solv}^{SASA}$. The term is computed as the product of an atom-specific solvation energy per surface area $\sigma_{i}^{SASA}$ and the atomic *SASA*_*i*_, summed over all atoms *i*.

(2) $$V_{solv}^{SASA}(\vec{r})=\sum_{i}\sigma_{i}^{SASA}\cdot SASA_{i}({\vec{r}}_{i})$$

In practice, $\sigma_{i}^{SASA}$ is either used in conjunction with a force field for vacuum simulations in which charged atom groups are parametrised as polar entities with nonzero total charge [11^••^,25^••^] or to model exclusively the nonpolar solute–solvent interactions in conjunction with a PB or GB model, in the following denoted as PB/SASA or GB/SASA (see below). To include long-range effects of the solvent on the solute's interior, the SASA solvation model has been further refined by a volume term [26] using a SASA/VOL switch function $g({\vec{r}}_{i})$

(3) $$V_{solv}^{VOL}(\vec{r})=\sum_{i}\sigma_{i}^{VOL}\cdot g({\vec{r}}_{i})\cdot \frac{4}{3}\pi R_{i}^{3}$$

and by recalibrated contributions of cavity formation and van der Waals interactions [27,28]. Several algorithms exist to compute the molecular SASA, spanning from exact to analytical approximations. An overview of the analysis of protein geometry can be found in Gerstein and Richards [29]. Fast methods for the SASA computation of proteins and nucleic acids are implemented in POPS [30,31] and POPSCOMP [32], a related application for the SASA analysis of biomolecular complexes. Instead of a SASA term, solely the volume of neighbour groups can be used as a measure of desolvation, as in the EEF1 [33^••^] and EEF1-SB models [12^••^].

(4) $$\Delta G_{i}^{sol}=\Delta G_{i}^{ref}-\sum_{j\neq i}f_{i}({\vec{r}}_{ij})\cdot VOL_{j}\text{,}$$

where $\Delta G_{i}^{ref}$ denotes the free energy of the fully solvated group *i* and the negative term the decrease in solvation free energy due to desolvation by neighbouring groups *j*.

### Poisson–Boltzmann

The Poisson equation relates the scalar electric potential *Φ* to the charge density *ρ*.

(5) $$\vec{\nabla }\cdot [\in (\vec{r})\vec{\nabla }\Phi (\vec{r})]=-\frac{\rho (\vec{r})}{\in_{0}}\text{,}$$

where *ɛ* is the local dielectric constant and *ɛ*_0_ the vacuum permittivity.

In practice the charge density may be obtained by integration of the Coulomb potential. The solution to the Poisson equation for a given charge density reads

(6) $$\Phi (\vec{r})=\frac{1}{4\pi \in_{0}\in }\int \frac{\rho (\vec{r})}{\left|\vec{r}\right|}dV(\vec{r})\text{.}$$

The Poisson–Boltzmann (PB) equation is a special case of the Poisson equation. At equilibrium the charge density is expected to follow a Boltzmann energy distribution

(7) $$\rho_{B}(\vec{r})=\sum_{i}c_{i}^{b}z_{i}qe^{-z_{i}q\Phi (\vec{r})/k_{B}T}\text{,}$$

where *c*_*i*_ denotes the bulk concentration of ion *i*, *z*_*i*_ its valence, *q* the unit charge, *k*_*B*_ Boltzmann's constant and *T* the temperature.

Eq. (5) applied to the charge density *ρ*_*B*_ yields the PB equation.

(8) $$\vec{\nabla }\cdot [\in (\vec{r})\vec{\nabla }\Phi (\vec{r})]=-\rho_{B}(\vec{r})-\sum_{i}c_{i}^{b}z_{i}qe^{-z_{i}q\Phi (\vec{r})/k_{B}T}$$

Being a differential equation, it is usually solved numerically. Given the exponential term is small enough ($e^{-z_{i}q\Phi (\vec{r})/k_{B}^{T}}\ll 1$), it can be approximated by the linear expression ($1-z_{j}q\Phi (\vec{r})/k_{B}T$), yielding the linearised PB equation.

(9) $$\vec{\nabla }\cdot [\in (\vec{r})\vec{\nabla }\Phi (\vec{r})]=-\rho_{B}(\vec{r})+\sum_{i}c_{i}^{b}z_{i}^{2}\frac{q^{2}\Phi (\vec{r})}{k_{B}T}$$

### Generalized Born

The Generalized Born (GB) equation was introduced by Still *et al.* [34^••^]. The Born model is a solution of the PB equation (see previous section) for a charge in the centre of an ideally spherical solute with radius *α* and internal dielectric *ɛ*_*int*_ in a solvent with dielectric *ɛ*_*ext*_:

(10) $$\Delta G_{solv}=-\frac{1}{2}\left(\frac{1}{\in_{\mathrm{int}}}-\frac{1}{\in_{ext}}\right)\frac{q^{2}}{\alpha }\text{.}$$

In the GB method a pseudo-ideal situation is emulated locally in non-ideal solutes (like biomolecules) by variation of *α*. By fixing the (computed or measured) value of Δ*G*_*solv*_ in Eq. (10), the undetermined radius *α* can be varied as control parameter to match the given Δ*G*_*solv*_. The resulting ‘effective Born radius’ *α* adjusts the local screening to an optimal value modulo approximations of the GB theory. The effective Born radii are used for the computation of the GB pair terms between charged atoms or atom groups *i* and *j*:

(11) $$\Delta G_{solv}=-\frac{1}{2}\left(\frac{1}{\in_{\mathrm{int}}}-\frac{1}{\in_{ext}}\right)\frac{q_{i}q_{j}}{\sqrt{r_{ij}^{2}+\alpha_{i}\alpha_{j}e^{-r_{ij}^{2}/4\alpha_{i}\alpha_{j}}}}\text{.}$$

Zhu *et al.* [35] implemented different GB methods and tested those in conjunction with the force fields GROMOS and OPLSAA.

Several GB methods have recently been implemented in the GROMACS package [36–38]. The GBn method of Mongan *et al.* [39^•^], which includes a volume correction for regions of interstitial solvent exclusion, is available in the AMBER package. An efficient parallelised GB/SASA algorithm has been implemented as a hybrid GPU/CPU application in the package NAMD [40].

### Combined PB/SASA, GB/SASA and related methods

Many biomolecular applications require a precision that demands combined PB/SASA and GB/SASA methods with complementary free energy contributions. In a recent study [41^•^] the methods HTC [37] and OBC [38] implemented in AMBER and the methods GBMV [42], GBSW [43] and FACTS (Fast Analytical Continuum Treatment of Solvation) [44^•^] implemented in CHARMM were compared.

## Parametrisation *via* force matching

Several options exist for the parametrisation of implicit solvents: first, matching the values of a reference method as in the parametrisation of the GB model to the more precise results of the PB model [36]; second, matching the known (free) energy differences of given reference states, for example the transfer free energies of small solutes [10^••^] or other relevant physico-chemical observables; third, reproducing known ensembles of the solute or the solvent, which implicitly reproduces the free energy landscape under the constraint that the conformational space has been represented realistically [25^••^,45^•^]; fourth, matching the solvation forces of molecular trajectories in explicit solvent [11^••^,12^••^,46].

The availability of large-scale simulation trajectories provides statistically meaningful force distributions that can be utilised to derive robust solvation parameters. A force-matching parametrisation (fourth above) is given in the following analytical formula for the determination of an atom-specific solvation parameter $\sigma_{i}^{SASA}$ for a SASA model [11^••^] (see also Eq. (2)):

(12) $$\sigma_{i}^{SASA}=-\frac{\partial A_{i}/\partial r_{i}}{|\partial A_{i}/\partial r_{i}{|}^{2}}\cdot \langle f_{i}^{\exp l}\rangle$$

The term ∂*A*_*i*_/∂*r*_*i*_ denotes the distance-dependent change of the exposed surface area and serves as projection direction for the explicit solvation force $\langle f_{i}^{\exp l}\rangle$. In practice, two sets of simulations are required to perform the parametrisation: a short Langevin simulation in implicit solvent to extract the ∂*A*_*i*_/∂*r*_*i*_ values and a long conformationally constrained simulation in explicit water to obtain the solvation forces, both using identical starting conformations to ensure correct projection geometry.

In an ensemble-matching parametrisation (third above), Bottaro *et al.* [46] used replica exchange MD simulations of the helical peptide (*AAQAA*)_3_ and the hairpin peptide *GB*1. Two parameters, a charge screening parameter and a backbone torsion term, were parametrised by minimising the Kullback–Leibler divergence between the configurational ensembles in explicit and implicit solvent. Because the Kullback–Leibler divergence is an information-theoretic metric, it is applicable to any type of distribution and therefore easily generalizable to other properties or combined properties.

An entirely different approach is the design of solvent-free coarse-grained PMFs, where the solvent contributions are included in the potential by integrating out solvent degrees of freedom during the parametrisation process [46,47]. The applied multiscale coarse-graining (MS-CG) method [48] fits observed forces in atomistic simulations by spline functions along a radial mesh connecting atoms. By employing a variational force-matching method, the residual between the effective atomistic and (to be parametrised) coarse-grained forces are minimised, thereby determining the linear fitting parameters of the coarse-grained force field.

## Use of energy terms on the basis of implicit solvation

We will focus here on the use of fast evaluation of the solvation energy term in protein dynamics, modelling, design and prediction.

## Protein modelling and design

Implicit solvent models like GB/SA coupled to energy minimisation have been proven to be more effective than MD in water [49] on a large-scale dataset with decoy models [50^•^]. Scheraga and co-workers applied their ECEPP05/SA force field combined to Monte-Carlo runs to discriminate native-like from non-native conformations in a set of decoys taken from the test set of the Rosetta@home all-atom decoys [51]. This approach identified successfully near-native conformations with a performance comparable with other existing physics-based scoring functions with computationally more expensive solvent models.

The Analytical Generalized Born plus Non-Polar (AGBNP) implicit solvent model has been shown to be effective in the prediction of the native conformations of medium-long loops (9–13 residues) for proteins with low sequence identity. The nonpolar solvation free energy predictor implemented in AGBNP with appropriate correction terms was crucial to attain the best prediction accuracy [49,52]. Recently, the group of Lavery [53] employed a simple geometrical measure, circular variance, to reflect residue burial (value range [0,1]) by the spatial distribution of neighbouring residues. This measure was used to build a very fast and effective model of protein solvation that was used to discriminate between native and decoy conformations.

In protein design implicit solvation is the method of choice for sidechain placement and mutant assessment. Lopes and Simonson [54] compared a parametrised SASA model [25^••^] to two GB models and showed that the former predicts the correct sign and order of magnitude of the stability change of mutants with a marginally higher error compared to the more accurate GB method. The same group developed a combined Coulomb/Accessible Surface Area (CASA) implicit solvent model, implemented it within the CHARMM19 force field and found that this combination was suitable for the computational engineering of ligands and proteins [55]. The group of Mayo has developed a Poisson–Boltzmann (FDPB) model ([56] and references therein) with a simplified surface term that is solely dependent on the identity and conformation of the protein backbone. This method has led to stable designed proteins *in vitro* and *in silico*.

## Protein dynamics: conformational equilibria and protein folding

Most of the implicit solvation models implemented in MD packages are routinely tested for their ability to reproduce conformational equilibria in explicit solvent and the cognate ensemble average properties (rmsd, rmsf, radius of gyration, exposed and buried atomic areas) [25^••^,26,32,33^••^,57]. Nevertheless, some challenging tests for implicit solvation can be found in seemingly simpler applications, like the reproduction of the conformational sampling of small dipeptides [58]. The group of McCammon reported the conformational sampling of alanine dipeptide in solution [45^•^] performed by means of the extended reference interaction site model (XRISM) integral equation theory. This approach is more detailed than a GB/SASA model, because the properties of the hydration structure can refine the PMF. The obtained Ramachandran plot is remarkably close to the one derived from simulations in explicit water and the typically observed solution minima basins are all densely populated. This model allows for the inclusion of interesting features like salt concentration effects, that is for the modelling of physiological conditions.

Significant progress has been made in the folding studies of small structures containing β-hairpins [59] or fragments from a series of very well characterised proteins [60] by means of replica exchange MD in GB/SASA solvent. A similar success has been achieved by Zagrovic and Pande within the Folding@home project [61]. Pande's group has also successfully folded [62] *ab initio* small fast-folder proteins using a GB/SASA/AMBERf99 parametrisation. In addition to revealing mechanisms of the folding process, these simulations sampled native-like and near-native conformations that show heterogeneity in their hydrophobic packing, mostly due to alternative side chain arrangements.

While β-sheets and β-hairpins seem to be easier to fold in implicit solvent, some inefficacies of the GB/SASA model in dealing with the folding equilibrium of *α*-helices have been highlighted by Nymeyer and García [63], where unphysical nucleation parameters were observed for implicit solvent models with respect to explicit water.

Despite the outlined progress, folding simulations in implicit solvent remain challenging for single helical structures or small protein folds that do not have a defined hydrophobic core. In a conceptually novel development, Duan *et al.* [64] have coupled discrete on-the-fly charge fitting schemes (Hydrogen Bond-specific Charge, HBC) with AMBER GB/SASA for the folding of a helical 17-residue peptide in a single 16 ns trajectory.

## Challenging applications: large-scale screening, binding free energy, nucleic acids and membranes

### Large-scale screening

Implicit solvation models play a central role in large-scale screening applications like the combinatorial assembly of protein-protein complexes and the estimation of the free energy of complexation. SASA calculations, if appropriately parametrised, provide a semi-quantitative estimate of the desolvation free energy upon protein complexation [32] and allow for interface prediction [65–67]. In docking score functions for protein complex prediction, the solvation term is often embedded in the electrostatic term [68], otherwise it is implemented as a separate implicit solvation term [33^••^] as in the Rosetta Docking program [69].

### Binding free energy

A challenging application for explicit and implicit solvation is the calculation of accurate binding free energies. The group of Essex has performed extensive comparisons between explicit solvation simulations and a GBSA implicit solvent model [70]. Despite some initial success [71], later results showed that explicit solvation leads to more accurate results [72].

Implicit-solvent based alchemical perturbation gives access to the configurational entropy, resulting in improved prediction of the binding free energy [73].

Overall, MD simulations in implicit solvent represent a viable route for medium to high-throughput computational drug discovery. In large-scale screening studies for structure-based drug design, even quite simple continuum models have been used successfully [74].

### Nucleic acids

Nucleic acids are a difficult target for the parametrisation of implicit solvent, due to their highly charged backbone and the required modelling of associated counter-ions. Most of the currently available implicit solvent models are unsuitable for MD simulations of structure and dynamics of nucleic acids. Instead, docking methods applied to RNA molecules have included implicit solvent models such as GBSA, PBSA and DOCK6 [75] and have devised useful strategies for effective structure-based RNA drug design.

An interesting review of the relationship between force field parametrisation (namely AMBER and CHARMM) and GB implicit solvent methods in simulating DNA and in extracting characteristic structural features is given by Gaillard and Chase [76^•^]. The work shows that GB methods have considerably improved over the last years and are approaching explicit solvation if judged by structural and dynamical features of the resulting ensembles. Developments in the design of GB/SASA and PB/SASA methods for RNA MD simulation by Liu *et al.* show that combining PB with a Langevin–Debye Model for the induced dielectric response of solvent and counter-ions improves the structural stability of the solute [77^••^]. The authors suggest that further improvement can be obtained by deriving the dielectric function with an iterative approach and by including explicitly more information about the topology and charge distribution of the solute.

### Membranes

One of the most challenging design problem for implicit solvent models is posed by the lipid-membrane environment, mostly due to the anisotropic nature of the medium and the associated contrasting hydrophobic/charged protein–lipid interactions depending on the relative positioning of the solute and the membrane. Ben-Tal, one of the pioneers, described the thermodynamics of an *α*-helix insertion while representing the membrane as a simple weakly dielectric slab [78]. Lazaridis’ IMM1 model [79] uses a generalization of the EEF1 solvation model [33^••^] to a membrane environment.

Implicit membrane models on the basis of the GB theory for ionic solvation have also been employed [43], extending previous work by the Brooks group [80], where a smoothing function was used to improve the numeric behaviour of the volume integration. Similarly, Spassov *et al.* [81^•^] considered the membrane to be part of the protein interior and analytic corrections to the Coulomb field term designed for soluble proteins were implemented. However, the assumption that the dielectric character of the membrane and of the protein are identical is rather unsatisfactory. Feig and coworkers developed a procedure to handle multiple dielectric environments [82^••^,83], the heterogeneous dielectric Generalized Born (HDGB) model, and applied it to implicit membrane modelling. Consequently, the membrane representation reproduces correctly the chemical heterogeneity of the membrane–water interface; a series of dielectric slabs, rather than just two regions, were used in the model. The PB method is used by many research groups to describe the membrane–protein association thermodynamics. A detailed discussion is provided in the review by Grossfield [84].

## Conclusions

Implicit solvation is gaining momentum in computational modelling and simulations of biomolecules due to the rapid progress in the development of multi-scale approaches and large-scale analysis of protein structures for use in protein design and targeted drug design. The diversity of models and implementations reflects the various requirements for the balance between time complexity of the computation and accuracy of the results. Details of the interactions between biomolecules and water carry valuable information for structural analysis [85,86]. A desirable feature of future implicit models solvent would be the possibility to preserve some of this information.

Working through the literature about implicit solvation, one cannot fail to note the absence of benchmark sets. It is common practice in most areas of computational biology to test novel methods on established benchmarks, for example the BaliBase benchmark for sequence alignment and the CASP targets for protein structure prediction. A benchmark set for implicit solvation would facilitate the cross-comparison between methods and provide a quantitative picture of the relationship between accuracy and computational expense. With the availability of repositories for MD trajectories, one could devise the development of reference test sets for controlled benchmarking of novel solvent models or parametrisations.

In the wake of genomics and proteomics, disease-related simulations and predictions can find a place as a diagnostic tool set, for example in the analysis of mutational effects and their influence on disease traits. Given the complexity of biomolecular structures and interactions, providing consistently reliable results within the diagnostic time constraints of a few days is beyond the scope of virtually any current computational method. It is however foreseeable that implicit solvent treatment will play a central role in such methods, because of their flexible applicability and tunable performance in multi-scale approaches to biomolecular characterisation and design.

## References and recommended reading

Papers of particular interest, published within the period of review, have been highlighted as:

• of special interest
•• of outstanding interest
